# Supplementary material for: On the physiological and structural contributors to the overall balance of excitation and inhibition in local cortical networks
Source: J Comput Neurosci. 2023 Oct 14;52(1):73–107. doi: 10.1007/s10827-023-00863-x (PMC11582336; doi:10.1007/s10827-023-00863-x)
Supplement: Supplementary file 1 — (pdf 1171 KB) [file 10827_2023_863_MOESM1_ESM.pdf]

On the Physiological and Structural Contributors to the Overall Balance of Excitation and Inhibition in Local Cortical Networks (Supplementary Material)

Farshad Shirani[1*] and Hannah Choi[1]

[1*]School of Mathematics, Georgia Institute of Technology, Atlanta, 30332, Georgia, USA.

*Corresponding author(s). E-mail(s): f.shirani@gatech.edu;
Contributing authors: hannahch@gatech.edu;

1. Verification of the key predictions of the mean-field model using the spiking network model

The key predictions of the mean-field model on the effects of variations in inhibitory synaptic decay time constants are verified in Section 4.5 of the main text using the spiking network model. Here, we perform similar analyzes to verify the key predictions of the mean-field model on the effects of variations in other network parameters. For each parameter, we simulate the spiking neuronal network at the same key dynamic states as described in the results shown in Figures 3–6, 8, and 9. We summarize the results obtained for each parameter in a separate figure, given below. For all simulations, the biophysical parameters of the spiking network are set as described in Section 3 of the main text, except for the parameters that are separately specified in each panel of each figure. In all simulations, the network receives Poisson-distributed background spike trains generated according to the description provided in Section 3 of the main text.

It should also be noted that, at some extreme values of the parameters, some adjustments are needed to be made on the spiking threshold potential of the neurons. The spiking activity of leaky integrate-and-fire neurons and their generalizations, such as the AdEx neurons we use in this paper, is dependent (to some extent) on the threshold potential chosen for their simplified spiking (resetting) mechanism. The curves of equilibrium values of electrochemical driving forces shown in the main text, which are calculated using the mean-field model, predict high levels of membrane depolarization or hyperpolarization in the network at some extreme parameter values. If the predicted mean membrane potential of neurons increases to values close to the baseline thresholds $\mathrm{V}_{\mathrm{E}}^{\mathrm{Thr}} = \mathrm{V}_{\mathrm{I}}^{\mathrm{Thr}} = -50$ mV, or even rises above this threshold, we observe hyper-synchronization and bursting in the activity of the spiking network. However, such activity is an artifact of the threshold used for the simplified spiking mechanisms of the neurons. In the opposite direction, significant decreases in the mean membrane potentials can result in a significant reduction in the firing activity of the spiking neurons, compared with the mean-field model, if no adjustment is made on the spiking thresholds. Therefore, guided by the predictions of the mean-field model on the values of mean membrane potentials, in some of the results presented below we have made adjustments on $\mathrm{V}_{\mathrm{E}}^{\mathrm{Thr}}$ and $\mathrm{V}_{\mathrm{I}}^{\mathrm{Thr}}$, compared with their baseline value, to avoid the artifacts of the resetting mechanisms of the neurons. When such adjustments are performed, the values chosen for $\mathrm{V}_{\mathrm{E}}^{\mathrm{Thr}}$ and $\mathrm{V}_{\mathrm{I}}^{\mathrm{Thr}}$ are reported in the corresponding figure.

In each panel of a figure shown below, a rastergram of the excitatory (red) and inhibitory (blue) spiking activity in the spiking neuronal network is shown on the left, for the last 2 seconds of the simulation. For visual clarity, only the activity of a randomly selected 10 percent subset of total neurons are illustrated. When the network does not present oscillatory bursting dynamics, the mean excitatory firing rate, the mean inhibitory firing rate, and the ratio of mean excitatory-to-excitatory synaptic conductance to mean inhibitory-to-excitatory synaptic conductance are given

on the right of each panel. For ease of reference, we recall that the values of these quantities at the baseline balanced state of the network, as calculated in Section 4.5 of the main text, are 1.13 Hz, 5.84 Hz, and 0.227, respectively. Additionally, the key observations made based on the spiking activity and conductance ratio in the network at the specified parameter value are also stated on the right of each panel.

The results show that, at a few extreme parameter values, the mean firing rates of the spiking network do not accurately match those of the mean-field model obtained at the same parameter value. See the discussion provided in Section 4.5 of the main text for possible reasons. At some other parameter values, however, there is a remarkable match between the firing rates and conductance ratios obtained from the two models. More importantly, in all cases, the key predictions made by the mean-field model on changes in the network balance and transitions to slow oscillatory regimes are confirmed by the activity of the spiking network. These predictions are summarized in Section 5 of the main text.

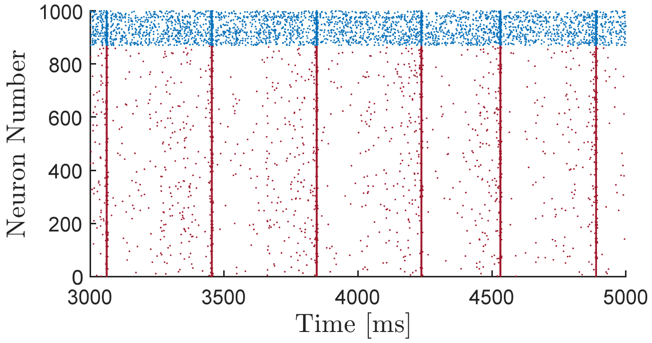

- $\tau_{\mathrm{EI}}^{\mathrm{Syn}} = \tau_{\mathrm{II}}^{\mathrm{Syn}} = 3$ ms
- **Observations:** Slow oscillatory bursting emerges in the network dynamics.

Fig. S1: **Effects of extremely low inhibitory synaptic decay time constants on the spiking activity of the network.** The value $\tau_{\mathrm{EI}}^{\mathrm{Syn}} = \tau_{\mathrm{II}}^{\mathrm{Syn}} = 3$ ms used here is beyond the range of values used in the mean-field model-based results shown in Figure 3 of the main text. The activity of the spiking network at the baseline value $\tau_{\mathrm{EI}}^{\mathrm{Syn}} = \tau_{\mathrm{II}}^{\mathrm{Syn}} = 8.3$ ms, as well as $\tau_{\mathrm{EI}}^{\mathrm{Syn}} = \tau_{\mathrm{II}}^{\mathrm{Syn}} = 6.5$ ms, are shown in Figure 11 of the main text.

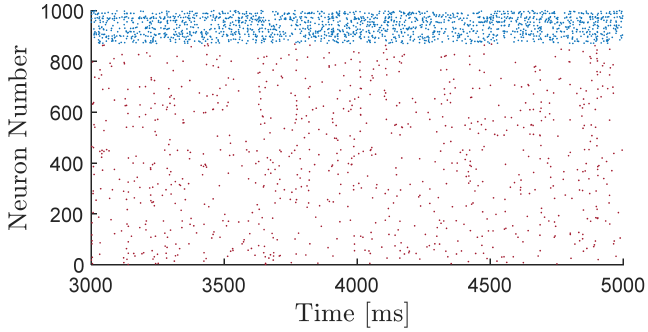

- $Q_{EI}^{Syn} = Q_{II}^{Syn} = 5$ nS
- $V_{E}^{Thr} = V_{I}^{Thr} = -45$ mV
- Mean excitatory firing rate = 0.51 Hz
- Mean inhibitory firing rate = 6.45 Hz
- $\frac{\text{Mean E-to-E synaptic conductance}}{\text{Mean I-to-E synaptic conductance}} = 0.41$
- **Observations:** Larger conductance ratio compared with its baseline value implies deviation in the network balance towards more excitation. However, there is still no transition in the network dynamics to a slow oscillatory bursting regime.

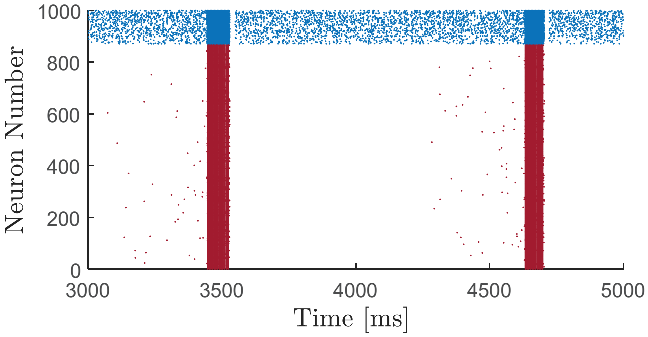

- $Q_{EI}^{Syn} = Q_{II}^{Syn} = 1$ nS
- $V_{E}^{Thr} = V_{I}^{Thr} = -45$ mV
- **Observations:** The network presents very high amplitude oscillatory bursting activity. The mean firing rate of excitatory and inhibitory populations during the bursting time intervals are approximately 160 Hz and 180 Hz, respectively.

Fig. S2: Effects of variations in synaptic quantal conductances on the spiking activity of the network. Corresponding observations made based on the mean-field model are given in Figure 4 of the main text.

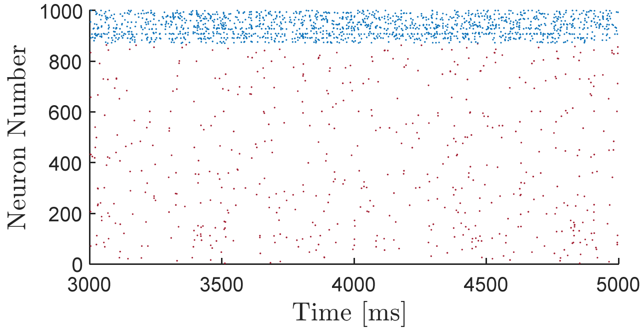

- $V_I^{Syn} = -60$ mV
- $V_E^{Thr} = V_I^{Thr} = -43$ mV
- Mean excitatory firing rate = 0.32 Hz
- Mean inhibitory firing rate = 5.73 Hz
- $\frac{\text{Mean E-to-E synaptic conductance}}{\text{Mean I-to-E synaptic conductance}} = 0.18$
- **Observations:** Reduced conductance ratio compared with its baseline value implies deviation in the network balance towards more inhibition.

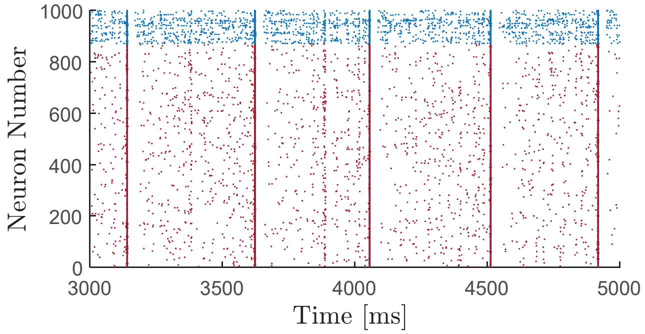

- $V_I^{Syn} = -86$ mV
- $V_E^{Thr} = V_I^{Thr} = -55$ mV
- **Observations:** Slow oscillatory bursting emerges in the network dynamics.

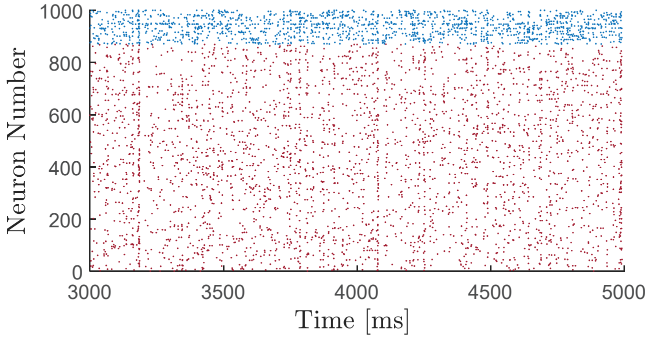

- $V_I^{Syn} = -100$ mV
- $V_E^{Thr} = V_I^{Thr} = -55$ mV
- Mean excitatory firing rate = 1.81 Hz
- Mean inhibitory firing rate = 5.85 Hz
- $\frac{\text{Mean E-to-E synaptic conductance}}{\text{Mean I-to-E synaptic conductance}} = 0.27$
- **Observations:** The network does not present oscillatory activity, while increased conductance ratio compared with its baseline value implies deviation in the network balance towards more excitation.

Fig. S3: **Effects of variations in synaptic reversal potentials on the spiking activity of the network.** Corresponding observations made based on the mean-field model are given in Figure 5 of the main text.

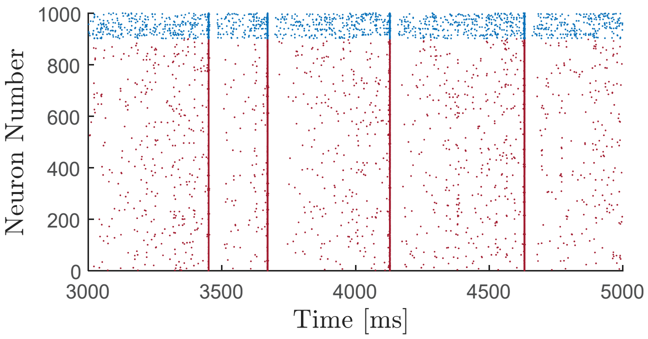

- $N_I/N = 0.1$
- **Observations:** Slow oscillatory bursting emerges in the network dynamics.

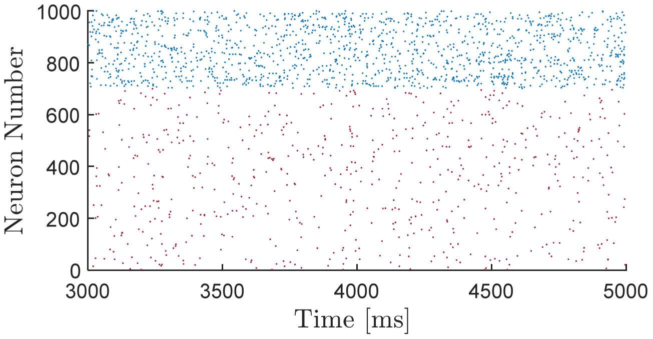

- $N_I/N = 0.3$
- Mean excitatory firing rate = 0.46 Hz
- Mean inhibitory firing rate = 2.50 Hz
- $\frac{\text{Mean E-to-E synaptic conductance}}{\text{Mean I-to-E synaptic conductance}} = 0.19$
- **Observations:** Reduced conductance ratio compared with its baseline value implies deviation in the network balance towards more inhibition.

Fig. S4: **Effects of variations in the ratio between the number of inhibitory and excitatory neurons on the spiking activity of the network.** Corresponding observations made based on the mean-field model are given in Figure 6 of the main text.

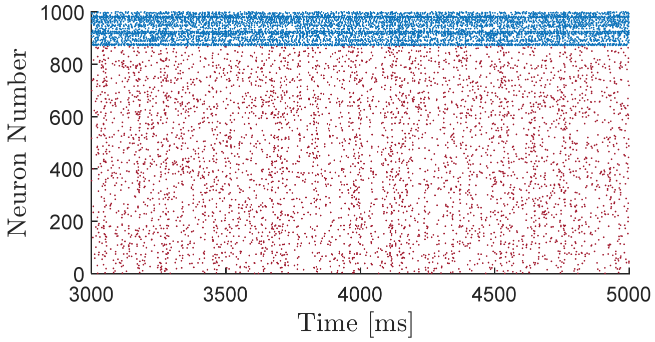

- $\mathrm{P_{EE} = P_{IE} = P_{EI} = P_{II} = 0.005}$
- Mean excitatory firing rate = 2.82 Hz
- Mean inhibitory firing rate = 28.14 Hz
- $\dfrac{\text{Mean E-to-E synaptic conductance}}{\text{Mean I-to-E synaptic conductance}} = 0.37$
- **Observations:** The network does not present oscillatory activity, while increased conductance ratio compared with its baseline value implies deviation in the network balance towards significantly more excitation.

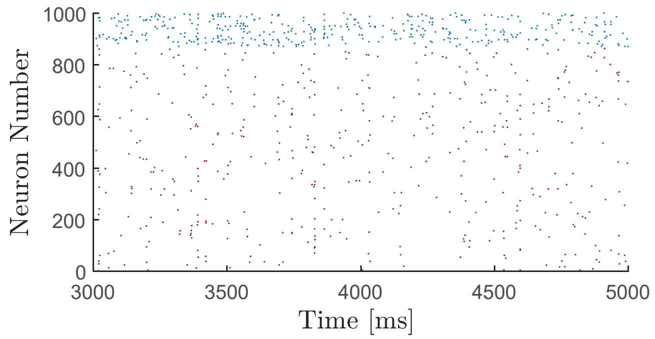

- $\mathrm{P_{EE} = P_{IE} = P_{EI} = P_{II} = 0.2}$
- Mean excitatory firing rate = 0.27 Hz
- Mean inhibitory firing rate = 1.59 Hz
- $\dfrac{\text{Mean E-to-E synaptic conductance}}{\text{Mean I-to-E synaptic conductance}} = 0.21$
- **Observations:** Reduced conductance ratio compared with its baseline value implies deviation in the network balance towards more inhibition.

Fig. S5: **Effects of variations in the overall network connectivity density on the spiking activity of the network.** Corresponding observations made based on the mean-field model are given in Figure 8 of the main text.

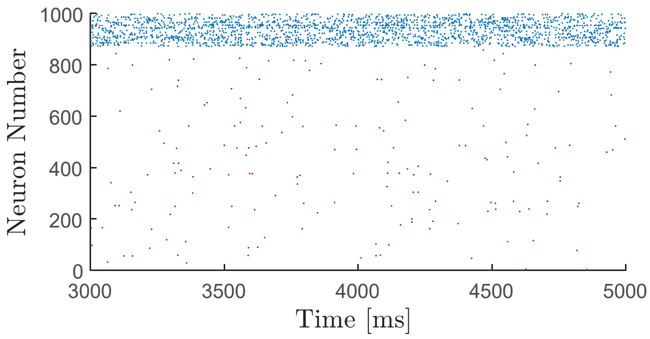

- $P_{II} = 0.03$
- Mean excitatory firing rate $= 0.10$ Hz
- Mean inhibitory firing rate $= 7.23$ Hz
- $\dfrac{\text{Mean E-to-E synaptic conductance}}{\text{Mean I-to-E synaptic conductance}} = 0.14$
- **Observations:** Reduced conductance ratio compared with its baseline value implies deviation in the network balance towards more inhibition.

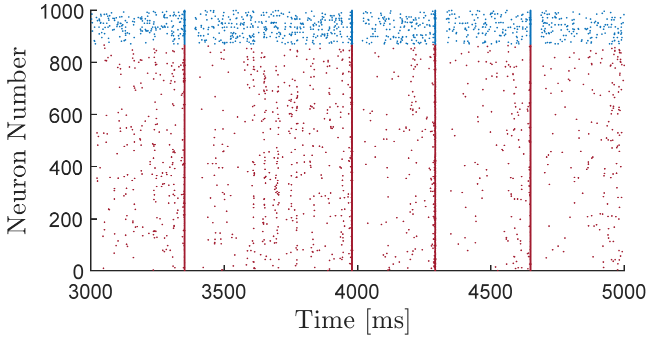

- $P_{II} = 0.06$
- $V_E^{Thr} = V_I^{Thr} = -45$ mV
- **Observations:** Slow oscillatory bursting emerges in the network dynamics.

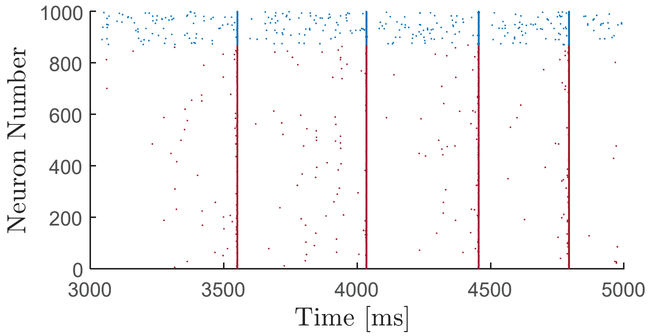

- $P_{II} = 0.11$
- $V_E^{Thr} = V_I^{Thr} = -35$ mV
- **Observations:** Slow oscillatory bursting emerges in the network dynamics. The difference between peak firing rates (at bursts) and lowest firing rates (during silence intervals) is more pronounced—as predicted by the large difference between the minimum and maximum values of firing rates on the limit cycles shown in Figures 9a and 9b of the main text.

Fig. S6: **Effects of variations in the inhibitory-to-inhibitory connection probability on the spiking activity of the network.** Corresponding observations made based on the mean-field model are given in Figure 9 of the main text.

2. Network dynamics during slow oscillatory bursting states

We provide a plausible explanation for the dynamic network activities that result in the emergence of slow oscillatory bursts in the network when the level of imbalance is critical. We base our analysis on the same results presented in Figure 11b of the main text, that is the results obtained when decay time constants were set at 6.5 ms for all inhibitory synapses, and as a results oscillatory activity emerged in the netwrok. Figure S7 shows the rastergram of spiking activity (for 10 percent of neurons), the mean adaptation current in the excitatory population, the mean excitatory membrane potential, and the mean inhibitory membrane potential obtained from our simulations.

Due to the level of imbalance in the network, towards more excitation, neurons gradually become more depolarized during the time intervals between the bursts. When the average level of depolarization in excitatory neurons reaches a sufficiently high level, subthreshold fluctuations in membrane potentials result in a relatively large number of excitatory neurons simultaneously become depolarized beyond their spiking threshold. As a result, a burst of spiking activity emerges in the excitatory neurons. Since the inhibitory population is also at a relatively high level of depolarization, the burst of excitatory spikes almost instantly results in a concurrent burst of activity in the inhibitory population.

A burst of excitatory activity initiates a surge of spike-triggered adaptation current in the excitatory population, which in turn causes a substantial level of hyperpolarization in the population. Consequently, the spiking activity of the excitatory population declines significantly right after the burst, which also results in a concurrent hyperpolarization and activity reduction in the inhibitory neurons as they lose a significant amount of their excitation.

The sharp activity reduction due to the hyperpolarization of the excitatory neurons after each burst causes the previously increased adaptation currents to decline, almost exponentially, which in turn allows the neurons to recover and become gradually depolarized due to the overall imbalance that exists in the network towards more excitation. This depolarization then results in another burst, and the sequence of events described above is repeated almost periodically, resulting in an oscillatory bursting activity in the network.

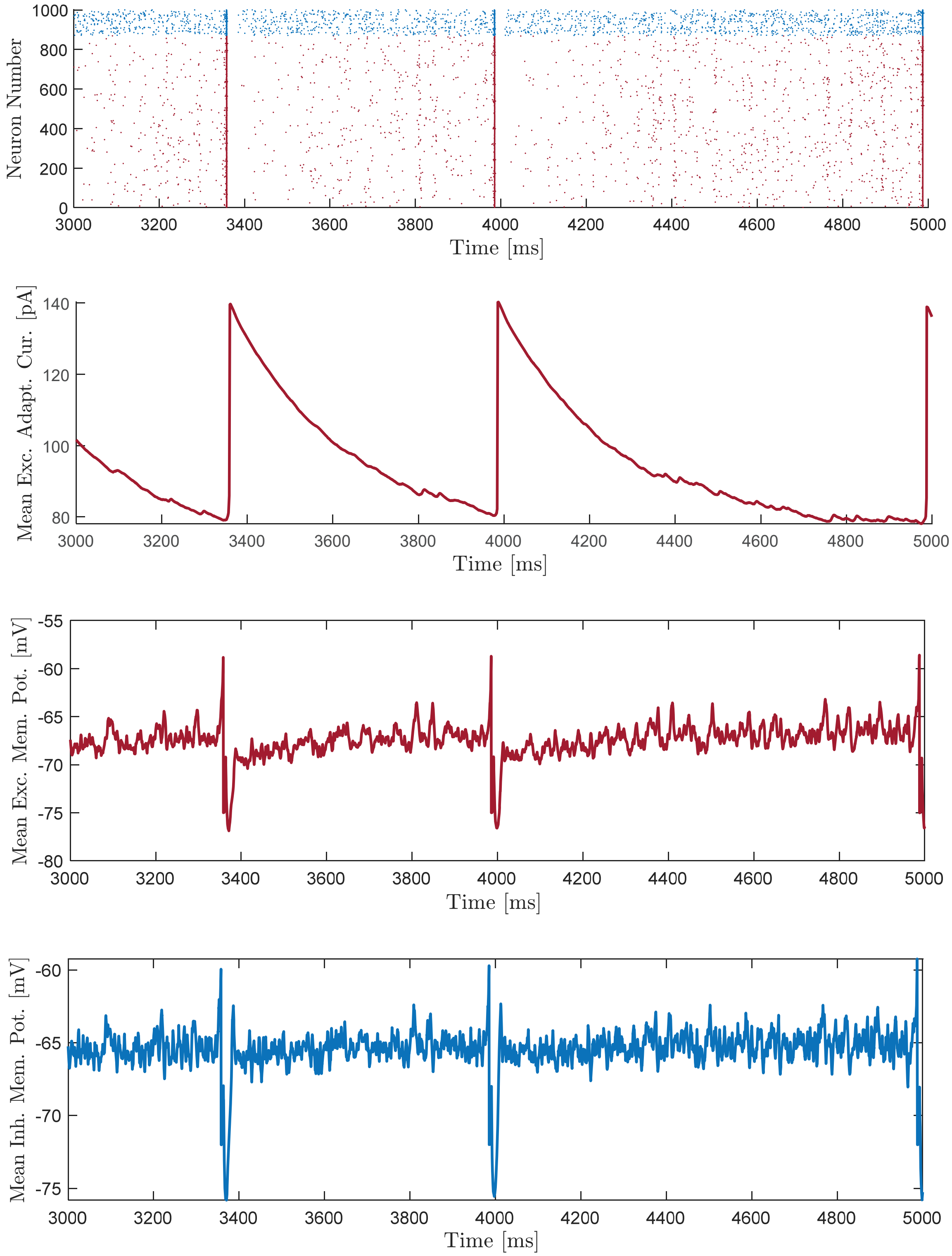

Fig. S7: **Spontaneous activity of the spiking neuronal network during the last 2 seconds of the slow oscillatory bursting state shown in Figure 11b of the main text.**
